# Single target acuity for moving targets in the common sunfish (*Lepomis gibbosus*)

**DOI:** 10.1242/bio.060455

**Published:** 2024-06-06

**Authors:** Marius Hoppe, Caroline Spratte, Frederike D. Hanke, Kenneth Sørensen

**Affiliations:** Institute for Biosciences, Neuroethology, University of Rostock, Albert-Einstein-Str. 3, 18059 Rostock, Germany

**Keywords:** Vision, Fish, Moving targets, Spatial resolution

## Abstract

The common sunfish (*Lepomis gibbosus*) likely relies on vision for many vital behaviors that require the perception of small objects such as detection of prey items or body marks of conspecifics. A previous study documented the single target acuity (STA) for stationary targets. Under many, if not most, circumstances, however, objects of interest are moving, which is why the current study tested the effect of the ecologically relevant parameter motion on sunfish STA. The STA was determined in two sunfish for targets moving randomly at a velocity of 3.4 deg/s. The STA for moving targets (0.144±0.002 deg) was equal to the STA for stationary targets obtained from the same fish individuals under the experimental conditions of this/the previous study. Our results contribute to a comprehensive understanding of fish vision, extending the large data set available on grating acuity.

## Teaser

**Summary:** This study reveals that the common sunfish's visual system perceives moving targets with equal precision as stationary targets.

## INTRODUCTION

In common sunfish (*Lepomis gibbosus*), vision is said to be involved in vital behaviors. In territorial and reproductive behavior, body patterns and the operculum dot (diameter approximately 10 mm) convey for example dominance to rivals or potential mates (Erickson, 1967; Stacey and Chiszar, 1978). During foraging, sunfish seem to be hunting visually (Miller, 1963; Colgan and Gross, 1977; Stacey and Chiszar, 1978) on prey as small as valve snails (*Valvata spp*., <2 mm; García–Berthou and Moreno–Amich, 2000).

In all behavioral contexts mentioned, sunfish need to perceive single targets. The visual resolution of single targets, called single target acuity (STA), measures how well a single target can be resolved from a uniform background. As STA is determined by contrast sensitivity (CS), objects smaller than suggested by retinal resolution, determining visual acuity (VA) assessed with gratings, can be perceived, provided their contrast to the background is high (Cronin et al., 2014; O'Carroll and Wiederman, 2014). With respect to common sunfish, STA for stationary targets was assessed as 0.144±0.022 deg (*N*=5) or 0.174±0.076 deg (*N*=6) including an individual with higher threshold in a behavioral experiment (Spratte et al., 2021). While this STA was obtained with full-contrast targets, real-world objects hardly have full contrast as processes such as scattering, and absorption degrade the object's contrast to background. When contrast was reduced from 0.98 to 0.41, sunfish STA deteriorated to 0.404±0.083 deg (*N*=6; Spratte et al., 2021). These results suggest a lower CS in common sunfish than in bluegill sunfish (*Lepomis macrochirus*) for which a CS function has previously been determined with gratings (peak at 0.4 cycles/deg, peak height of 30, cut-off frequency at approximately 5 cycles/deg; Northmore et al., 2007).

In all above mentioned contexts, moving objects and their perception are relevant: sunfish hunt a variety of moving prey, and motion also contributes to the detection of conspecifics, or predators (Clark and Keenleyside, 1967). A moving target is eliciting a successive change in brightness in neighboring photoreceptors, and motion information is extracted by a comparison of the photoreceptors' signals over time (see, e.g. Cronin et al., 2014). The most famous model proposed to explain motion detection/direction determination is the Hassenstein-Reichardt detector (Hassenstein and Reichardt, 1956; Reichardt, 1961; or a variant by Barlow and Levick, 1965), and experimental evidence, particularly from flies, support this model's processing stages (for reviews see Mauss et al., 2017; Borst and Groschner, 2023). In fish, motion vision was suggested to be mediated by double cones (Boehlert, 1978; Siebeck et al., 2014), although their contribution is still debated, and generally the direction selective retinotectal system (for review see Damjanović et al., 2023).

In this study, we set out to determine whether and how sunfish STA is affected when the object is moving. Different results for stationary and moving STA could be obtained due to different properties of the processing pathways for stationary and moving objects from the retina to the brain (for primates: Livingstone and Hubel, 1988). CS, forming the basis for STA, changes with target movement for example in birds and humans (Robson, 1966; Kelly, 1979; Burr and Ross, 1982; Haller et al., 2014). In birds, comparing CS for stationary versus moving stimuli, target movement mostly improved the CS at low, but far less at high spatial frequencies. The comparable CS for high spatial frequencies for stationary and moving stimuli might have resulted in an equal STA for stationary and moving targets in budgerigars (Chaib et al., 2019; Chaib et al., 2021). As the comparison of CS for stationary and moving stimuli revealed interspecific differences, we were interested in comparing STA for stationary and moving targets for another vertebrate species, the common sunfish, in a behavioral experiment for comparison (another vertebrate tested for stationary/moving STA is the harbor seal; Sandow and Hanke, 2024). While numerous studies on fish vision tested grating acuity (Douglas and Hawryshyn, 1990; Caves et al., 2017) and, to our knowledge, three studies assesed STA (Ben-Simon et al., 2012; Temple et al., 2013; Champ et al., 2014), a comparison between moving and stationary STA has not been made in any fish species yet. Our STA study thus extends previous research on the resolving capacities of fish eyes.

### RESULTS AND DISCUSSION

**Fig. 1.**
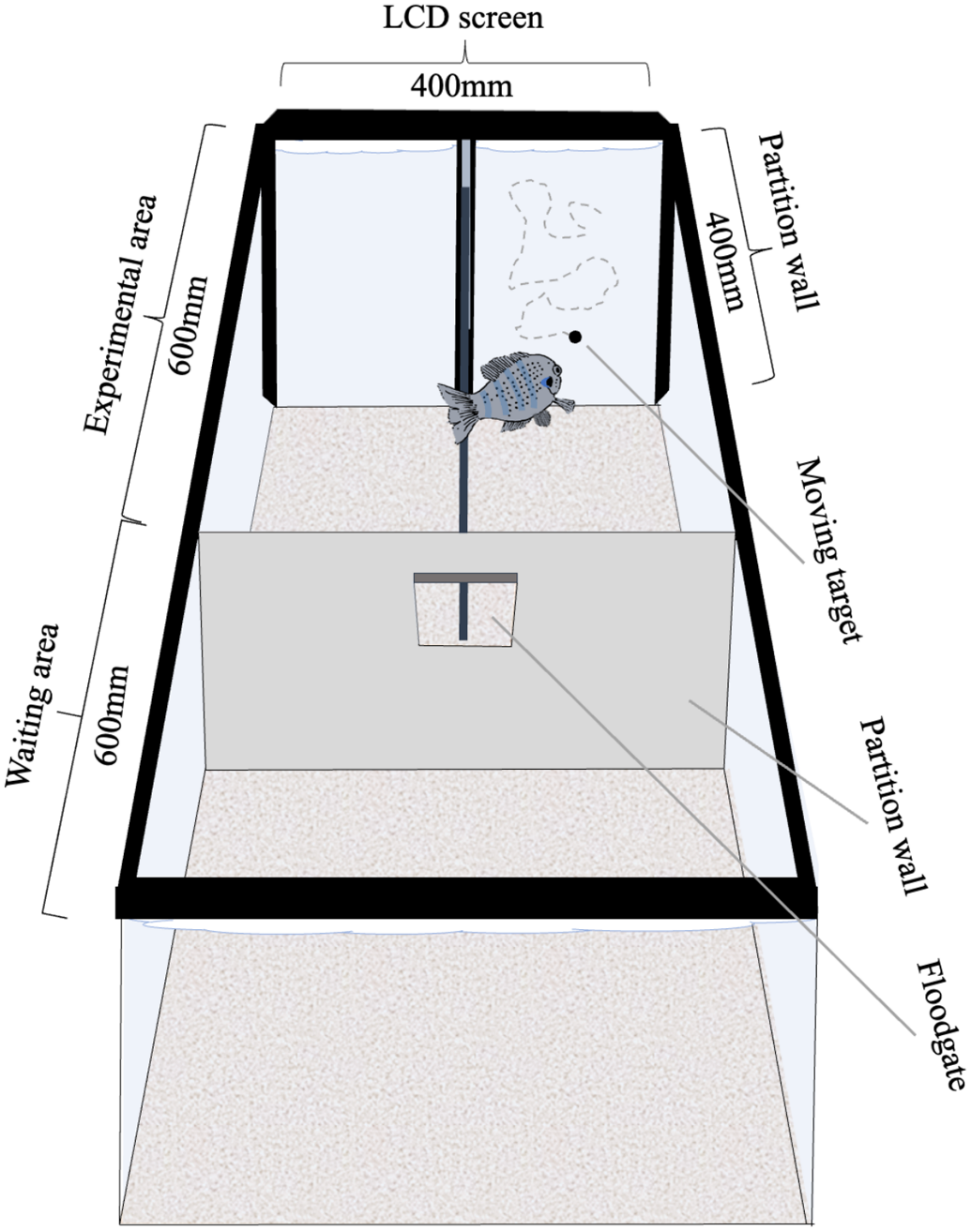
**Experimental setup for STA determination for moving targets with common sunfish.** The fish started each session in the waiting area. The floodgate could be opened and closed between holding area and experimental area allowing the fish to enter the experimental area at the beginning of each trial. At the far end of the experimental area, an LCD screen was attached to the aquarium from outside. The area in front of the monitor was divided into two decision areas by a partition wall. The fish had to swim into the decision area at which end the positive stimulus (moving target; here: at the end of the right decision area) was presented on the monitor. The monitor remained white in the alternative decision area (here: at the end of the left decision area).

All three fish successfully transferred the experimental paradigm directly, meaning within the three sessions required to fulfill the learning criterion, from stationary (Spratte et al., 2021) to moving targets (Table 1; Fig. S2); thus, the moving target elicited a robust behavioral response. In the generalization phase of training (Table 1), the fish only needed 2-5 sessions more than minimally required to fulfill the learning criterion to complete training stages 4 and 5. Two fish (Hannah and Lina) successfully completed threshold determination after we had determined four and six STA thresholds (Fig. 1). We did not observe an improvement in STA thresholds over time as reported in previous STA studies most likely as the two fish had already participated in STA training with stationary targets.

**Table 1.**
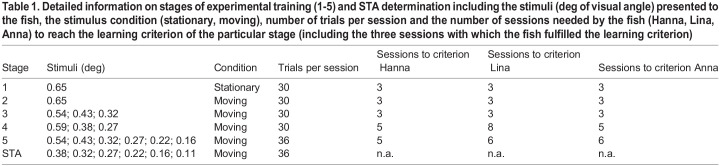
Detailed information on stages of experimental training (1-5) and STA determination including the stimuli (deg of visual angle) presented to the fish, the stimulus condition (stationary, moving), number of trials per session and the number of sessions needed by the fish (Hanna, Lina, Anna) to reach the learning criterion of the particular stage (including the three sessions with which the fish fulfilled the learning criterion)

The final STA values for moving targets for the two fish amounted to 0.145±0.011 deg and 0.143±0.003 deg, respectively (Table 2). There was no significant difference between the moving STA of the two fish (two-way Student's *t*-test, *P*>0.05). Thus, the average STA of two common sunfish for full contrast targets moving at a velocity of 3.4 deg/s amounted to 0.144±0.002 deg. Fish Anna stopped cooperating after we had determined a single STA threshold of 0.126 deg. We were not able to obtain the minimally required three STA thresholds (see STA determination) and thus cannot report a final STA value for fish Anna.

**Table 2.**
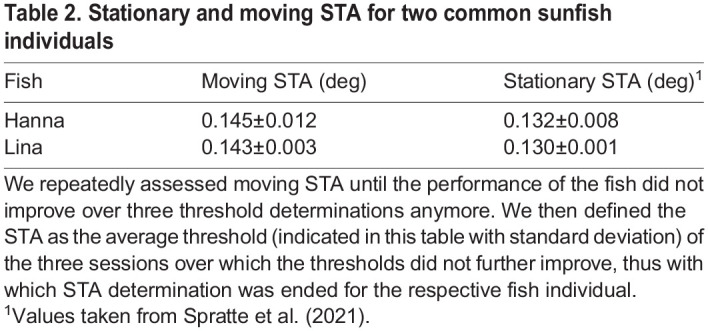
Stationary and moving STA for two common sunfish individuals

Two aspects need to be stressed: first, we can only report final STA values for two old common sunfish. However, there are numerous reasons why we consider our results reliable and informative: the fish behaved normally throughout the experiment, the STAs of these individuals were very similar, the single threshold we obtained from a third fish was in the same range, and stationary STAs determined in more, namely six common sunfish generally did not deviate strongly interindividually (Spratte et al., 2021). Furthermore, the big advantage of our data set is that it allows us to directly compare performances on individual level assessed under the same experimental conditions.

Second, we examined the common sunfish's STAs in only one experimental condition. In line with Spratte et al. (2021), documenting a decrease of stationary STA when decreasing contrast between object and background, single or multiple target parameters such as the velocity at which or the way the single object is moving as well as its shape might lead to different results such as to a difference between stationary and moving STA. Results deviating from the results of the two sunfish STA studies (current study and Spratte et al., 2021) might be obtained if the sunfish's visual system was adapted to specific object features or the better the experimental conditions resemble the conditions found in the sunfish's natural habitat.

The average moving STA was not significantly different from the average stationary STA for full contrast targets amounting to 0.144±0.022 deg (two-way Student's *t*-test, *P*>0.05; Table 2; Spratte et al., 2021), although the comparison of the STA values for the two fish individuals suggested stationary STA to be slightly better by 0.01 deg than moving STA (Table 2). A comparable correspondence of stationary and moving STAs was reported for budgerigars (Chaib et al., 2019; Chaib et al., 2021). With respect to the physiology behind STA, we would thus expect that a systematic analysis of common sunfish CS would reveal that motion has only a slight or even no effect on the sunfish's CS for high spatial frequencies comparable to the CS of budgerigars (Haller et al., 2014) not only for targets, but maybe in general. The determination of CS functions under different stimulus conditions in common sunfish can be the topic of future behavioral studies. While, in our opinion, behavioral experiments best describe what an organism can actually see (Douglas and Hawryshyn, 1990), other methodological approaches, such as electrophysiology, need to be chosen when attempting to understand the physiological basis of CS, visual resolution, or the characteristics of processing pathways in general.

The sunfish's STAs compare well with the STAs of other organisms, neglecting methodological differences of the different studies for the moment. To our knowledge, among the fish, STA has only been determined for archerfish (*Toxotes jaculatrix*; Ben-Simon et al., 2012) a predatory fish, and triggerfish (*Rhinecanthus aculeatus*; Champ et al., 2014), a generalist. The sunfish's STAs fall within the lower range of the archerfish's STA (between 0.075-0.15 deg; Ben-Simon et al., 2012), whereas they are superior to the triggerfish's STA (0.4-0.8 deg; Champ et al., 2014). This comparison might suggest predatory fish to have a higher STA than generalists, which would be in line with a speculation by Caves et al. (2017). These authors speculated that predatory fish could profit from increased sensitivity to serve the task of spotting a prey evading the predator as under water the prey would decrease in contrast rapidly due to strong attenuation. However, a larger data set on fish STA would be needed to draw well-grounded conclusions. A large data set could then help to correlate eye size or ecological parameters with STA (see analysis by Caves et al., 2017 for fish VA) to assess for example whether functional properties of the visual system reflect the respective ecological niches or lifestyles of fish (Collin and Pettigrew, 1988).

The field of research focusing on vision in fish might generally profit from combining data sets on STA as well as on VA assessed with gratings. Data from behavioral grating VA determination in common sunfish suggest VA to be similar to STA (M. Muck, University of Rostock, unpublished data). Grating VA of a closely related sunfish, the bluegill sunfish, was assessed as 3.4-5 cycles/deg and is thus also within the range of the common sunfish's STA (Northmore et al., 2007; Spratte et al., 2021). Although grating VA and STAs should be compared with caution due to different underlying processing mechanisms (O'Carroll and Wiederman, 2014), the combination of both measures of resolution, that might not always coincide as in common sunfish, might give a more comprehensive picture of vision in fish as we currently have.

Our STA values allow the calculation of viewing distances (see extensive discussion in Spratte et al., 2021), which would amount to a distance of 70-120 cm for objects with high contrast to background. Comparing this viewing distance to reactive distance measures from the bluegill sunfish (Werner and Hall, 1974; O'Brien et al., 1976; Breck and Gitter, 1983), our STA values suggest a much larger viewing distance. This discrepancy most likely cannot be explained by interspecific differences in visual abilities as all studies on vision in sunfish so far demonstrate similar visual abilities. In our opinion, reactive distance approaches do not necessarily determine the ultimate limits of prey detection as the sunfish – or generally any species tested with respect to reactive distances – might show a measurable reaction to a prey item only at distances closer to the item than suggested by sensory perception. Reactive distances might alternatively be indicative of the distance at which a predator starts considering the final attack.

In reactive distance approaches and generally under natural conditions, viewing distances alone cannot entirely define the ‘active space’ (Caves et al., 2018) of a species. Information from other sensory modalities might extend the limits imposed by vision with respect to what details can be perceived and from what distance. Motion information could additionally be picked up by the lateral line system; it can also replace the visual system entirely when vision is temporally impaired or impossible such as in the blind cavefish in which the lateral line system contributes to prey detection (Yoshizawa et al., 2010). Green sunfish (*Lepomis cyanellus*) preferably used information from their lateral line system instead of vision in multimodal hunting conditions (Janssen and Corcoran, 1993). Thus, future experiments should focus on unimodal stimulation of the lateral line system the same way as we presented optic stimuli in isolation but could subsequently address interesting questions on multisensory integration by stimulating sunfish multimodally to mimic the usually multimodal natural conditions encountered during complex behaviors such as foraging.

## MATERIALS AND METHODS

### Experimental animals

We included three female common sunfish (13-14 years of age), thus all sunfish that were available, in our training. The sunfish had been obtained from an aquarium supplier years before this study. During the course of the study, training had to be stopped with one fish (fish Anna) due to uncooperativeness. All fish had taken part in the first STA study (Spratte et al., 2021). Each fish was kept in a freshwater aquarium (120×40×55 cm; 240 l), with coarse-grained substrate and vegetation on one side of the aquarium. The water temperature was kept constant at 20-24°C, and the water parameters were regularly monitored using JBL ProScan water analysis strips (JBL GmbH & Co. KG, Neuhofen, Germany). The fish were fed red mosquito larvae (*Chironomidae*) during experimental sessions. Usually, two sessions were conducted per day, 5-6 days per week.

All experiments were carried out in accordance with the European Communities Council Directive of September 22, 2010 (63/210/EU). The individuals used in the study were not subject to pain, suffering or injury; therefore, no approval or notification was required. Maintenance was approved by local authorities (Landesministerium für Landwirtschaft, Lebensmittelsicherheit und Fischerei Mecklenburg-Vorpommern).

### Experimental setup

For the experiments, the aquarium was divided into a waiting area and an experimental area, separated by a gray partition wall with a floodgate that could be opened from outside (Fig. 1; same setup as in Spratte et al., 2021). In the experimental area, an LCD screen (resolution: 1024×768px, 1px=0.377 mm, model no. 1907Fpf, Dell Inc., Round Rock, TX, USA) was attached to the short side of the aquarium from outside. The screen served to present the stimuli. A partition wall (400×3×550 mm) divided the experimental area in front of the screen into a left and right decision area. To prevent secondary cue giving from the experimenter and to avoid any events from outside the aquarium interfering with the experimental procedure in general, the aquarium was completely covered from all sides with black film and white panels. Only the top of the aquarium remained open allowing to handle and reward the fish according to its behavior. The aquarium was illuminated from above with two aquarium lamps (background luminance 132 cd/m²; MultiLux LED NATURE 6500K, LED DAY 9000K, JUWEL Aquarium, Rotenburg, Germany).

### Stimuli

The positive stimulus consisted of one circular black target with adjustable diameter on a white stimulus field measuring 150×380 mm; it was tested against a homogenously white stimulus field defined as negative stimulus. The Weber contrast of the target to the background was 0.98 and was calculated on the basis of luminance measurements conducted with a luminance meter (LS-110, Konica Minolta, Langenhagen, Germany). The target was moving semi-randomly within a 7.0×7.0 cm movement area in the center of the stimulus field. The velocity of the target was set to 3.4 deg/s corresponding to a medium locomotion velocity of potential prey organisms of the common sunfish, such as *Daphnia magna* (swimming velocity 0.5-9.9 deg/s; Table S1). All stimuli were programmed in and presented with MatLab (R2018b, The Mathworks, Natick, MA, USA) and the Psychophysics Toolbox (Pelli and Vision, 1997; Kleiner et al., 2007; m-file adapted from an m-file written by S. Chaib).

The moving single target was presented pseudo-randomly (Gellermann, 1933) in either the left or the right stimulus field. For each single target (Table 1), its diameter was assessed on photos taken by an underwater camera (WP5 2020 outdoor Smartphone, OUKIEL, Guan Lan, Silicon Valley, CA, USA). The diameter was determined from the luminance profile, obtained in ImageJ (1.53n, Rasband, National Institutes of Health, Bethesda, MD, USA) and exported to OriginPro 2018b (b9.5.5.409, OriginLab Corporation, Northampton, MA, USA), as full width at half amplitude. On each photo, the diameter of the target was measured three times and pixel values were converted in mm values with the help of a scale. The visual angle (in deg; Table 1) was calculated for a viewing distance of 400 mm, corresponding to the length of the partition wall.

### Experimental procedure

Each trial started by opening the floodgate for the fish. After entering the experimental area, the fish had to swim into the decision area, at which end the positive stimulus was presented on the LCD screen, in line with a two-alternative-forced-choice experiment. A decision was considered to have been made when the fish crossed the edge of the partition wall until its distal end of the operculum. For a correct decision, the fish was rewarded with one to two red mosquito larvae provided through a syringe. After the feedback, the monitor turned black, the fish had to enter the waiting area, and the floodgate was closed. If the fish made an incorrect response, the screen turned black immediately, and no reward was given.

### Training phase

Training was started (stage 1; Table 1) with a stationary single target to commence training with a stimulus the fish were familiar with from the first STA study (Spratte et al., 2021). We then added motion to this target (stage 2). In stages 3-5, we asked the fish to generalize the experimental procedure and thus introduced new target diameters and increased the variation to three (stages 3 and 4) and six (stage 5) target diameters per session. Additionally, these stages served to determine the target diameters to be used for STA determination. A training phase was terminated once the fish reached the learning criterion defined as a performance of ≥80% correct choices, slightly better than predicted by a significance level of *P*=0.01 (χ² test), in three consecutive sessions.

### STA determination

**Fig. 2.**
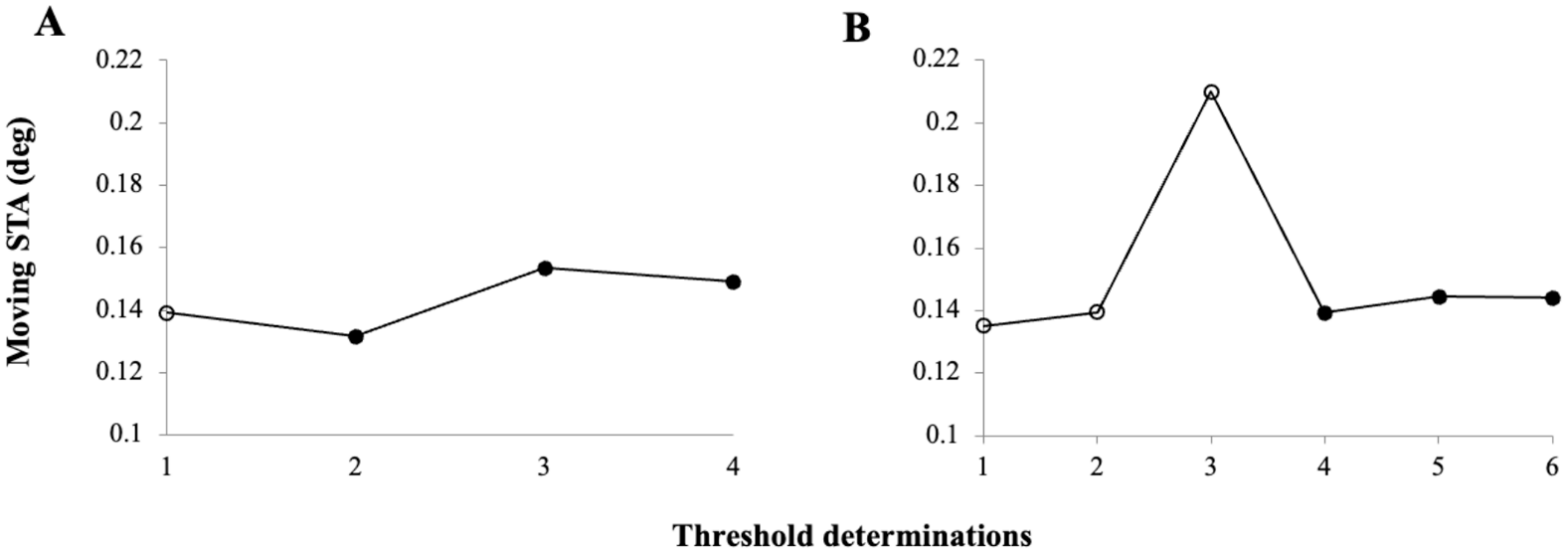
**Moving STA thresholds (in deg) determined for two common sunfish individuals A fish Hanna and B fish Lina from the psychometric functions (see Fig. S1) over the course of data collection.** STA thresholds for moving targets were repeatedly assessed as previous work (see for example Chaib et al., 2019, Spratte et al., 2021) had shown STA thresholds to decrease over threshold determination, meaning with experience of the experimental animals. Data collection was ended once the threshold value did not improve over three threshold determinations (solid dots). In this study, the moving STA was defined as the mean value of the three threshold determinations (solid dots) with which the criterion was fulfilled. The final STA value (Table 2) was derived by averaging the STA thresholds of these last three threshold determinations. Please note that we are not plotting the single STA threshold we obtained in the third fish, fish Anna; however, we report this value in the manuscript for completeness.

According to the method of constant stimuli, six targets (Table 1), four targets with putatively suprathreshold and two with putatively subthreshold diameters/visual angles, were chosen to determine the STA. Each target was presented 30 times over five sessions, and the number of correct choices for each target were determined. The resulting psychometric function plotting correct choices as a function of visual angle (Fig. S1) was analyzed to determine the 75% threshold by linear interpolation of the last suprathreshold and first subthreshold value. We repeatedly assessed the moving STA (Fig. 2) as STA values often improve over threshold determinations (see for example Chaib et al., 2019; Spratte et al., 2021) and defined the moving STA as the average of the last three STA determinations during which the performance did not improve as in Spratte et al. (2021).

## Supplementary Material

10.1242/biolopen.060455_sup1 Supplementary information
