# Supplementary material for: Single target acuity for moving targets in the common sunfish (Lepomis gibbosus)
Source: Biol Open. 2024 Jun 6;13(6):bio060455. doi: 10.1242/bio.060455 (PMC11179713; doi:10.1242/bio.060455)
Supplement: Supplementary information [file biolopen-13-060455-s1.pdf]

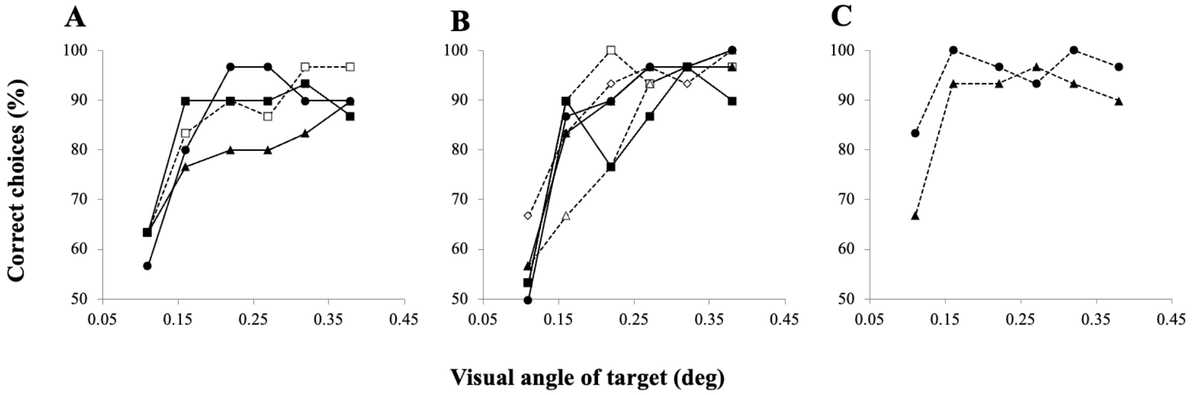

Fig. S1. Psychometric functions showing correct choices (in %) as a function of the visual angle of the target (in deg) for three common sunfish individuals **A** fish Hanna, **B** fish Lina, and **C** fish Anna. Each data point represents the performance of the sunfish averaged over 36 presentations of the respective target. From the psychometric function, the STA threshold was determined as the 75% threshold by linear interpolation of the last suprathreshold value and the first subthreshold value. Solid lines and markers represent the last three threshold determinations with which the performance had reached a plateau (Supplement Fig.2); consequently, threshold determination was terminated thereafter. Please note, with fish Anna, we could only obtain a single STA threshold. During the second threshold determination, we were not able to calculate a threshold as the fish's performance did not fall below 75% correct choices. Unfortunately, the fish stopped cooperating thereafter.

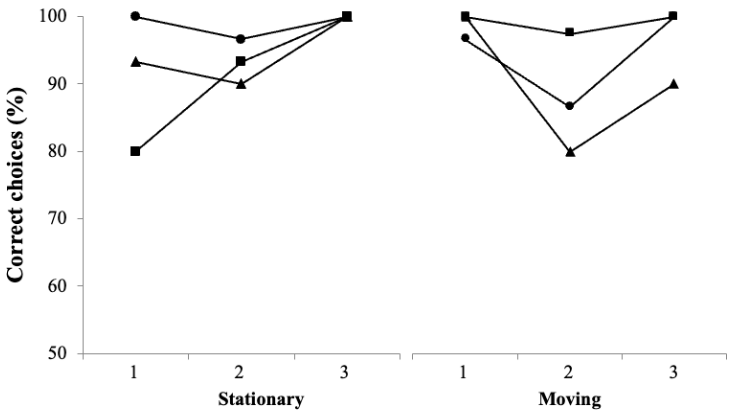

Fig. S2. Learning curves (stage 1 and stage 2, see Tab.1 in manuscript) with correct choices (in %) for three common sunfish individuals **A** fish Hanna (square), **B** fish Lina (triangle) and **C** fish Anna (dot). During these two training stages, a target with a visual angle of 0.65 deg was presented. All fish successfully transitioned from stationary to moving targets and fulfilled the learning criterion of a performance ≥80% correct choices in three consecutive sessions for threshold determination directly.

Biology Open • Supplementary information

Table S1. Swimming velocity (in mm/s) of *Daphnia magna* as documented in a selected number of publications (full references in footnote). Swimming velocity is given in mm/s from which the swimming velocity in deg/s was calculated. For the calculation of swimming velocity in deg/s, mm was converted in deg of visual angle as seen from a distance of 400 mm (length of the partition wall in our experimental setup) by the common sunfish of our study

	Velocity (mm/s)	Velocity (deg/s) from a distance of 400mm
(Bownik *et al.* 2017)	50	7
(Bownik *et al.* 2019)	5.5	0.8
(Hylander *et al.* 2014)	7.5-40	1.1-5.7
(Langer *et al.* 2019)	15-20	2.1-2.9
(Larsson & Kleiven 1996)	3.5-30	0.5-4.3
(Serra *et al.* 2018)	70	9.9
(Tesson & Sha 2020)	5-27	0.7-3.8
(Wickramarathna *et al.* 2014)	15-40	2.1-3.4

Bownik, A., M. Pawlocik and N. Sokolowska. 2017. Effects of neonicotinoid insecticide acetamiprid on swimming velocity, heart rate and thoracic limb movement of *Daphnia magna*. Polish Journal of Natural Sciences 32:481-493.

Bownik, A., B. Slaska, J. Bochra, K. Gumieniak and K. Galek. 2019. Procain penicillin alters swimming behaviour and physiological parameters of *Daphnia magna*. Environmental Science and Pollution Research 26:18662-18673.

Hylander, S., M. T. Ekvall, G. Bianco, X. Yang and L.-A. Hansson. 2014. Induced tolerance expressed as relaxed behavoural threat response in millimetre-sized aquatic organisms. Proceedings of the Royal Society B: Biological Sciences 281: 20140364.

Langer, S. M., L. C. Weiss, M. T. Ekvall, G. Bianco, L.-A. Hansson and R. Tollrian. 2019. A three-dimensional perspective of Daphnia's swimming behavior with and without predator cues. Limnology and Oceanography 64:1515-1525.

Larsson, P. and O. T. Kleiven. 1996. Food search and swimming speed in *Daphnia*. Pages 375-387 *in* P. H. Lenz, D. K. Hartline, J. E. Purcell and D. L. Macmillan eds. *Zooplankton: sensory ecology and physiology.* Gordon and Breach Publishers, Amsterdam.

Serra, T., A. Barcelona, M. Soler and J. Colomer. 2018. Daphnia magna filtration efficiency and mobility in laminar to turbulent flows. Science of the Total Environment 621:626-633.

Tesson, S. V. M. and Y. Sha. 2020. Population connectivity, dispersal, and swimming behavior in *Daphnia*. Ecology and Evolution 11:2873-2885.

Wickramarathna, L. N., C. Noss and A. Lorke. 2014. Hydrodynamic trails produced by Daphnia: size and energetics. PloS One 9:e92383.

Biology Open • Supplementary information
